# Comparison of the clinical presentation across two waves of COVID-19: a retrospective cohort study

**DOI:** 10.1186/s12879-022-07413-3

**Published:** 2022-05-03

**Authors:** Henriette Nørmølle Buttenschøn, Vibeke Lynggaard, Susanne Gundersborg Sandbøl, Eva Natalia Glassou, Annette Haagerup

**Affiliations:** 1 NIDO | Centre for Research and Education, Gødstrup Hospital, Hospitalsparken 25, 7400 Herning, Denmark; 2 Department of Clinical Medicine, Aarhus University, Palle Juul-Jensens Boulevard 82, 8200 Aarhus N, Denmark; 3 Cardiovascular Research Unit, Department of Cardiology, NIDO | Centre for Research and Education, Gødstrup Hospital, Hospitalsparken 25, 7400 Herning, Denmark; 4 Department of Quality, NIDO | Centre for Research and Education, Gødstrup Hospital, Hospitalsparken 25, 7400 Herning, Denmark

**Keywords:** Clinical characteristics, Coronavirus, COVID-19, Critical disease, Exposure, Laboratory data, Mortality, Period, Wave, Source of infection

## Abstract

**Background:**

Only a few studies have performed comprehensive comparisons between hospitalized patients from different waves of COVID-19. Thus, we aimed to compare the clinical characteristics and laboratory data of patients admitted to the western part of Denmark during the first and second waves of COVID-19 in 2020. Furthermore, we aimed to identify risk factors for critical COVID-19 disease and to describe the available information on the sources of infection.

**Methods:**

We performed a retrospective study of medical records from 311 consecutive hospitalized patients, 157 patients from wave 1 and 154 patients from wave 2. The period from March 7 to June 30, 2020, was considered wave 1, and the period from July 1st to December 31, 2020, was considered wave 2. Data are presented as the total study population, as a comparison between waves 1 and 2, and as a comparison between patients with and without critical COVID-19 disease (nonsurvivors and patients admitted to the intensive care unit (ICU)).

**Results:**

Patients admitted during the first COVID-19 wave experienced a more severe course of disease than patients admitted during wave 2. Admissions to the ICU and fatal disease were significantly higher among patients admitted during wave 1 compared to wave 2. The percentage of patients infected at hospital decreased in wave 2 compared to wave 1, whereas more patients were infected at home during wave 2. We found no significant differences in sociodemographics, lifestyle information, or laboratory data in the comparison of patients from waves 1 and 2. However, age, sex, smoking status, comorbidities, fever, and dyspnea were identified as risk factors for critical COVID-19 disease. Furthermore, we observed significantly increased levels of C-reactive protein and creatinine, and lower hemoglobin levels among patients with critical disease.

**Conclusions:**

At admission, patients were more severely ill during wave 1 than during wave 2, and the outcomes were worse during wave 1. We confirmed previously identified risk factors for critical COVID-19 disease. In addition, we found that most COVID-19 infections were acquired at home.

## Background

Coronavirus disease 2019 (COVID-19) is a global pandemic with more than 236 million confirmed cases worldwide as of October 10, 2021 [1]. Since COVID-19 on March 11, 2020, was declared a pandemic by the World Health Organization, several countries have observed a wave pattern in the number of COVID-19 cases with increased numbers of cases in the high peak months of 2020 [2–4]. The first confirmed COVID-19 patient in Denmark was reported on February 27, 2020, and the disease soon became widely spread in the Danish community [5, 6]. The current population of Denmark is approximately 5.8 million. Denmark has as of October 10, 2021, 2669 confirmed deaths related to COVID-19 and 363,900 confirmed cases of which 17,553 has been hospitalized [6]. The daily number of diagnosed and hospitalized Danish patients has changed over time, and the outbreak in Denmark has likewise been observed as two waves in 2020 [6]. In line with other countries and as a consequence of the increasing number of COVID-19 cases in wave 1, the Danish government introduced a series of prevention measures that gradually were removed during the Danish summermonths and reintroduced in the third quarter of 2020. Most Danish and international studies of the clinical characteristics have primarily focused on patients from the first wave of COVID-19 [7–13]. Only a few studies have performed comprehensive investigations of the similarities and differences between hospitalized patients in different wave periods [2, 3, 14–16]. To our knowledge no Danish study has previously compared patients from the first and second waves.

Thus, the primary aim of this study was to compare and identify differences in the demographic, clinical, and laboratory characteristics of patients hospitalized in the western part of Denmark during the first and second wave periods of COVID-19 in 2020. Furthermore, we aimed to identify risk factors for critical COVID-19 disease and describe the given information on the sources of infection 14 days prior to infection.

## Methods

### Study design and participants

This retrospective study included consecutive hospitalized patients regardless of the duration of hospitalization. The study was conducted at the Regional Hospital West Jutland (RHWJ).

To be included in the study, patients tested positive at least once with a SARS-COV-2 polymerase chain reaction (PCR) result. All patients were either inhabitants or stayed in the geographical area served by the hospital at the time of hospitalization. Included patients were admitted from March 7 to December 31, 2020. None of the patients had received a vaccination for COVID-19 prior to admission.

### Data sources

Electronic medical records from inpatients with a positive SARS-COV-2 PCR result were obtained and reviewed. In total, five trained reviewers participated in the review process of the medical records. All medical records were reviewed independently and cross-checked by a second reviewer.

### Measures

We obtained information on demographical data, lifestyle information, comorbidities, vital signs at admission, disease symptoms, treatments, initial laboratory data, and clinical outcomes from the medical records. Furthermore, the medical records were reviewed to obtain information on the sources of infection, e.g., (1) infection at home, (2) infection by a person outside home, (3) infection at hospital, (4) infection at an institution, or (5) infection by an unknown source.

The initial laboratory data from the hospital presentation included C-reactive protein (CRP), lactate dehydrogenase, lymphocyte counts, leucocyte counts, thrombocyte counts, hemoglobin, alanine aminotransferase, bilirubin, alkaline phosphatase, creatinine, potassium, and sodium.

### Study definitions

We defined the two wave periods of the COVID-19 outbreak in Denmark in 2020 as follows: the first period from March 7 to June 30, 2020, and the second period from July 1st to December 31, 2020.

Nonsurvivors and patients admitted to the intensive care unit (ICU) were classified as having critical disease. Fatal disease was defined as death during admission or death within 30 days after the diagnosis. The length of hospitalization was defined as the actual number of days admitted to the hospital. Hospital admissions were defined as *long hospitalizations* if the duration of the admission was eight days or longer. Comorbidity was defined as the presence of at least one underlying medical condition. A total of 13 comorbidities were included the study: Asthma, stroke or transient ischemic attack, coronary heart disease/ischemic heart disease, psychiatric disorder, diabetes mellitus, hypertension, chronic bronchitis and chronic obstructive pulmonary disease, cancer, rheumatoid arthritis and connective tissue diseases, neurological diseases, osteoarthritis, metabolic diseases, nephrological and urological diseases. Cancer was defined to include all types of cancer. Neurological diseases included epilepsy, sclerosis, neuralgias, and parkinson's disease. Metabolic diseases included hyperthyroidism and hypothyroidism. Nephrological and urological diseases included all diseases in the kidneys and the urinary tract. Initial laboratory data were defined as the first available laboratory test result at hospital presentation. Recorded symptoms (fever, runny nose, cough, sore throat, shortness of breath/dyspnea, headache, muscle aches/myalgia, diarrhea, malaise, nausea, vomiting, and tiredness) denote if the patient had experienced any of the symptoms at least once during their COVID-19 infection.

### Statistical analyses

Data were managed using electronic data capture tools (REDCap) hosted in the Central Denmark Region [17, 18].

Data analyses were mainly descriptive. Continuous variables are presented as medians and interquartile ranges (IQRs). Categorical variables are presented as counts (N) and percentages. The Mann–Whitney U test (Wilcoxon rank-sum test) was used for comparisons between continuous variables, and the chi-squared test or Fisher´s exact test was used to compare differences in categorical variables. Missing data was not imputed. A p-value below 0.05 was considered significant. All analyses were performed using Stata 17.

## Results

### Baseline characteristics

**Fig. 1:**
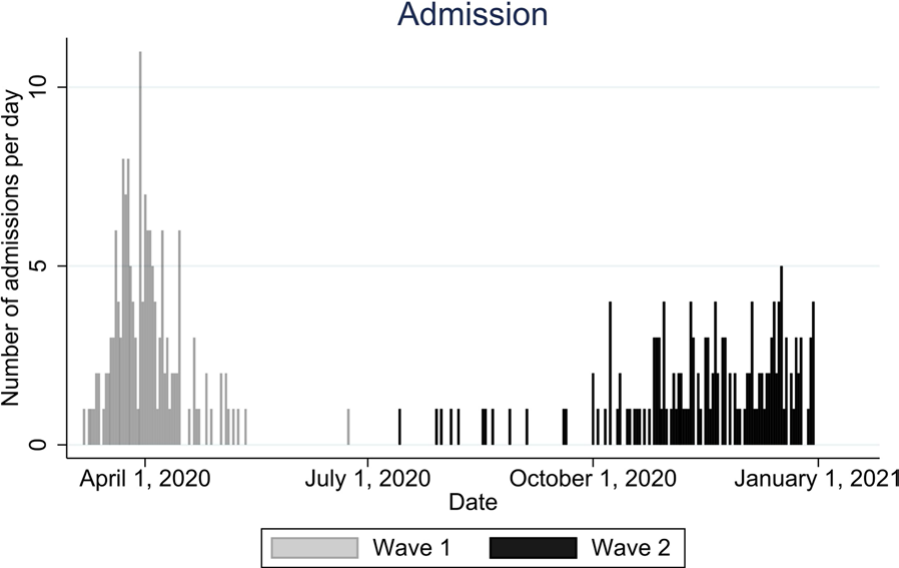
Illustration of the number and dates of hospital admissions during waves 1 and 2

In total, 311 COVID-19 patients were admitted to the hospital. One hundred fifty-seven patients were admitted during the first wave and 154 were admitted during the second wave. The distribution of daily admitted patients from March 7 to December 31, 2020, is illustrated in Fig. 1, and details of the baseline characteristics are shown in Table 1. The median age for all patients was 64 years (interquartile range (IQR): 50–77 years).

**Table 1:** Presentation and comparisons of baseline characteristics

		Wave			Critical disease		
	All patients (N = 311)	Wave 1 (N = 157)	Wave 2 (N = 154)	p-value	Yes (N = 54)	No (N = 257)	p-value
Socio demographics and lifestyle information							
Age, Median (IQR) (N = 314)	64 (50–77)	65 (51–78)	63.5 (47–77)	0.27	74.5 (67–83)	61 (47–75)	** < 0.0005**
Age distribution N/total N (%)				0.49			** < 0.0005**
0–9	10/311 (3.22)	2/157 (1.27)	8/154 (5.19)		0/54	10/257 (3.89)	
10–19	4/311 (1.29)	2/157 (1.27)	2/154 (1.30)		0/54	4/257 (1.56)	
20–29	11/311 (3.54)	4/157 (2.55)	7/154 (4.55)		1/54 (1.85)	10/257 (3.89)	
30–39	22/311 (7.07)	9/157 (5.73)	13/154 (8.44)		0/54	22/257 (8.56)	
40–49	29/311 (9.32)	17/157 (10.83)	12/154 (7.79)		1/54 (1.85)	28/257 (10.89)	
50–59	52/311 (16.72)	25/157 (15.92)	27/154 (17.53)		4/54 (7.41)	48/257 (18.68)	
60–69	60/311 (19.29)	33/157 (21.02)	27/154 (17.53)		13/54 (24.07)	47/257 (18.29)	
70–79	63/311 (20.26)	34/157 (21.66)	29/154 (18.83)		18/54 (33.33)	45/257 (17.51)	
80–89	48/311 (15.43)	23/157 (14.65)	25/154 (16.23)		11/54 (20.37)	37/257 (14.40)	
90 +	12/311 (3.86)	8/157 (5.10)	4/154 (2.60)		6/54 (11.11)	6/257 (2.33)	
Gender N/total N (%)				0.95			**0.003**
Female	150/311 (48.23)	76/157 (48.41)	74/154 (48.05)		16/54 (29.63)	134/257 (52.14)	
Male	161/311 (51.77)	81/157 (51.59)	80/154 (51.95)		38/54 (70.37)	123/257 (47.86)	
Smoking N/total N (%) (N = 195)				0.94			** < 0.0005**
Present smoker	19/195 (9.74)	11/106 (10.38)	8/89 (8.99)		6/46 (13.04)	13/149 (8.72)	
Previous smoker	79/195 (40.51)	43/106 (40.57)	36/89 (40.45)		29/46 (63.04)	50/149 (33.56)	
Never smoker	97/195 (49.74)	52/106 (49.06)	45/89 (50.56)		11/46 (23.91)	86/149 (57.72)	
Body Mass Index (BMI), Median (IQR) (N = 198)	27.17 (22.86–32.05)	27.20 (23.41–31.95)	27.10 (22.77–32.05)	0.75	26.44 (22.71–31.35)	27.33 (23.41–32.27)	0.44
Comorbidities							
Comorbidity N/total N (%)	248/311 (79.44)	121/157 (77.07)	127/154 (82.47)	0.24	51/54 (94.44)	197/257 (76.65)	**0.003**
Comorbidity burden N/total N (%)				0.28			** < 0.0005**
Single comorbidity	67/311 (21.54)	29/157 (18.47)	38/154 (24.68)		3/54 (5.56)	64/257 (24.90)	
Multiple comorbidities	181/311 (58.20)	92/157 (58.60)	89/154 (57.79)		48/54 (88.89)	133/257 (51.75)	
Specific comorbidities N/total N (%)							
Asthma	24/311 (7.72)	9/157 (5.73)	15/154 (9.74)	0.21	5/54 (9.26)	19/257 (7.39)	0.58
Stroke or transient ischemic attack	23/311 (7.40)	6/157 (3.82)	17/154 (11.04)	**0.017**	5/54 (9.26)	18/257 (7.00)	0.57
Coronary heart disease/Ischemic heart disease	59/311 (18.97)	28/157 (17.83)	31/154 (20.13)	0.67	19/54 (35.19)	40/257 (15.56)	**0.002**
Psychiatric disorder	37/311 (11.90)	17/157 (10.83)	20/154 (12.99)	0.60	5/54 (9.26)	32/257 (12.45)	0.65
Diabetes mellitus	53/311 (17.04)	27/157 (17.20)	26/154 (16.88)	1	13/54 (24.07)	40/257 (15.56)	0.16
Hypertension	102/311 (32.80)	58/157 (36.94)	44/154 (28.57)	0.12	28/54 (51.85)	74/257 (28.79)	**0.001**
Chronic bronchitis and chronic obstructive pulmonary disease	45/311 (14.47)	27/157 (17.20)	18/154 (11.69)	0.20	16/54 (29.62)	29/257 (11.28)	**0.001**
Cancer	42/311 (13.50)	31/157 (19.75)	11/154 (7.14)	**0.001**	17/54 (31.48)	25/257 (9.73)	** < 0.0005**
Rheumatoid arthritis and connective tissue diseases	21/311 (6.75)	13/157 (8.28)	8/154 (5.19)	0.37	6/54 (11.11)	15/257 (5.84)	0.23
Neurological disease (eg. epilepsy, sclerosis)	22/311 (7.07)	16/157 (10.19)	6/154 (3.90)	**0.045**	9/54 (16.67)	13/257 (5.06)	**0.006**
Osteoarthritis	20/311 (6.43)	16/157 (10.19)	4/154 (2.60)	**0.009**	8/54 (14.81)	12/257 (4.67)	**0.012**
Metabolic diseases (eg. hyperthyroidism and hypothyroidism)	31/311 (9.97)	25/157 (15.92)	6/154 (3.90)	** < 0.0005**	10/54 (18.52)	21/257 (8.17)	**0.041**
Nephrological and urological diseases	54/311 (17.36)	35/157 (22.29)	19/154 (12.34)	**0.025**	19/54 (35.19)	35/257 (13.62)	**0.001**

The distributions of baseline characteristics are presented for all patients. Baseline characteristics were compared between patients admitted in waves 1 and 2 and patients with and without critical COVID-19 disease

*IQR* interquartile range, *N* number

Approximately 80% of all patients had at least one comorbidity, and more than half of all patients had multiple comorbidities. The most common comorbidity was hypertension. Further analyses showed that patients with at least one comorbidity were older than those with no comorbidity (p < 0.0005, median age 69 years (IQR: 56–79 years) versus 44 years (IQR: 31–57 years)).

No differences in age, sex, smoking, and body mass index (BMI) were observed between the patients hospitalized in RHWJ in wave 1 compared to wave 2. However, we observed differences in the frequencies between the specific comorbidities, e.g., more patients with metabolic disease and cancer were observed during the first COVID-19 wave than during the second wave.

The median age increased significantly among patients with critical COVID-19 disease. Furthermore, significantly more males and present or previous smokers were observed among patients with critical disease. Patients with critical disease were also more likely to suffer from one or more comorbidities.

### Vital signs and laboratory findings

The vital signs at admission and initial laboratory tests are shown in detail in Table 2. Due to clinical practice, information on vital signs and laboratory findings was not available for all patients.

**Table 2:** Presentation and comparisons of vital signs and laboratory results at hospital presentation

		Wave			Critical disease		
	All patients (N = 311)	Wave 1 (N = 157)	Wave 2 (N = 154)	p-value	Yes (N = 54)	No (N = 257)	p-value
Vitals at hospital presentation							
Temperature ≥ 38, N/total N (%)	118/299 (39.46)	73/150 (48.67)	45/149 (30.20)	**0.001**	29/54 (53.70)	89/245 (36.33)	**0.018**
Oxygen saturation, median (IQR) N = 298	96 (94–98)	96 (94–98)	97 (95–99)	**0.03**	95 (93–97)	97 (95–99)	**0.0001**
Respiratory rate, median (IQR) N = 297	20 (18–24)	20 (18–24)	20 (18–24)	0.23	24 (20–28)	20 (18–24)	**0.001**
Systolic blood pressure, median (IQR) N = 290	134 (122–151)	136 (122–155)	133 (122–147.5)	0.35	131 (118–145)	135 (122–153)	0.13
Diastolic blood pressure, median (IQR) N = 288	76 (68–87)	75.5 (67–87)	76.5 (70–87)	0.36	71 (60–81)	77 (70–87)	**0.0053**
Pulse, median (IQR) N = 297	87 (75–98)	86 (74–97)	88 (75–99)	0.34	83.5 (73–100)	87 (75–98)	0.68
Laboratory findings at hospital presentation							
C-reactive protein (CRP) (mg/l; normal range < 8), median (IQR), N = 268	39 (12.35–95)	41.4 (16.9–105.5)	32.5 (7.65–91)	0.084	76.5 (33–131)	31.5 (10.1–80)	**0.0001**
Lactate Dehydrogenase (U/l; normal range 105–205), median (IQR), N = 175	236 (194–315)	245 (196–328)	235 (194–300)	0.52	251.5 (209.5–336)	234 (192–310)	0.17
Lymphocytes (× 10^9^/l; normal range 1.3–3.5), median (IQR), N = 254	0.98 (0.69–1.41)	1.02 (0.69–1.38)	0.95 (0.69–1.41)	0.72	0.91 (0.58–1.3)	1.03 (0.7–1.41)	0.26
Leucocytes (× 10^9^/l; normal range 3.5–10), median (IQR) N = 270	5.9 (4.6–8.4)	6.05 (4.7–9.1)	5.75 (4.55–7.75)	0.085	6.5 (4.5–9.1)	5.9 (4.6–8.2)	0.21
Thrombocytes (× 10^9^/l; normal range 165–400 females, 145–350 males), median (IQR), N = 234	199 (153–255)	210 (155–276)	193 (142–245)	0.071	183 (136–258)	204 (155–248)	0.37
Hemoglobin (mmol/l; normal range females 7.3–9.5, males 8.3–10.5), median (IQR), N = 264	8.2 (7.3–8.9)	8.2 (7.2–8.8)	8.3 (7.6–9)	0.11	7.6 (6.8–8.6)	8.3 (7.6–9)	**0.0094**
Alanine aminotransferase (U/l; normal range 10–45), median (IQR), N = 212	25 (18–37.5)	26.5 (18–40.5)	23.5 (17.5–34)	0.31	21 (16–40)	25 (18–37)	0.51
Bilirubin (µmol/l; normal range 5–25), median (IQR), N = 199	9 (7–11)	9 (7–11)	9 (7–12)	0.41	9 (7–12.5)	9 (7–11)	0.67
Alkaline phosphatase (U/l; normal range 35–105), median (IQR), N = 232	75.5 (62–94)	76.5 (63.5–94.5)	72.5 (60–93.5)	0.33	74 (56–98)	76 (63–93)	0.74
Creatinine (µmol/l, normal range 45–90), median (IQR), N = 267	81 (64–108)	82.5 (70.5–111)	79 (62–100)	0.065	94 (80–128)	79 (62–100)	**0.0001**
Potassium (mmol/l; normal range 3.5–4.6), median (IQR), N = 265	3.9 (3.6–4.2)	3.8 (3.6–4.2)	3.9 (3.6–4.2)	0.90	3.9 (3.6–4.3)	3.8 (3.6–4.1)	0.21
Sodium (mmol/l; normal range 137–145), median (IQR), N = 266	138 (136–140)	138 (136–140)	138 (136–140)	0.21	137 (135–139)	138 (136–140)	0.09

The distributions of vital signs and laboratory results are presented for all patients. Vital signs and laboratory results were compared between patients admitted in waves 1 and 2 in addition to patients with and without critical COVID-19 disease

The normal range for laboratory data (adults > 18 years) are give in parenthesis

Bold p-values specify significance < 0.05

*IQR* interquartile range, *N* number

In the total study population, we observed elevated levels of inflammatory markers. The median levels of CRP (39 IQR:12.35–95) and lactate dehydrogenase (236 IQR:194–315) were above the normal ranges (< 8 mg/l and 105–205 U/l, respectively). Furthermore, the median lymphocyte count (0.98 × 10^9^ IQR:0.69–1.41) was below the normal range (1.3–3.5 × 10^9^/l).

Patients admitted during the wave 1 were more likely to present with a higher temperature and a lower oxygen saturation at admission. No significant differences in laboratory data between patients hospitalized in wave 1 and wave 2 were observed. However, we observed a tendency toward higher CRP and creatinine levels in addition to higher leucocyte and thrombocyte counts in wave 1 compared to wave 2.

Patients with critical disease were more likely to present with a higher temperature and respiratory rate in addition to lower oxygen saturation and diastolic blood pressure at admission. Higher CRP and creatinine levels and a lower hemoglobin levels were observed among patients with critical disease compared to other patients. Among patients with critical disease, further analyses showed a significantly higher creatinine level among those with a fatal outcome than among those surviving a critical disease course (p = 0.048, median creatinine level 107 µmol/l (IQR: 85–159) versus 83 µmol/l (IQR: 76–124)).

### Treatment, outcomes, and symptoms

Details on treatment and outcomes, in addition to symptoms during infection are shown in Table 3. Approximately 10% (N = 31) of all admitted patients were treated in the ICU. Likewise, 10% (N = 31) died during admission or within 30 days after their diagnosis. Twenty-five percent (N = 8) of the nonsurvivors were admitted to the ICU. Oxygen supplementation was administered in more than half of the patients, and mechanical ventilation was administered in 7% of the patients. The median time from onset of disease symptoms to hospital admission was four days (IQR: 2–8), and the median length of hospitalization for all patients was also four days (IQR: 0–9). The most common disease symptoms were fever, followed by dyspnea and cough.

**Table 3:** Presentation and comparisons of treatments, outcomes, and symptoms

		Wave			Critical disease		
	All patients (N = 311)	Wave 1 (N = 157)	Wave 2 (N = 154)	p-value	Yes (N = 54)	No (N = 257)	p-value
Treatment and outcomes							
Critical disease, N/total N (%)	54/311 (17.20)	45/157 (28.66)	9/154 (5.84)	** < 0.0005**			
Fatal disease, N/total N (%)	31/311 (9.97)	25/157 (15.92)	6/154 (3.90)	** < 0.0005**	31/54 (57.41)		
ICU care, N/total N (%)	31/311 (9.97)	25/157 (15.92)	6/154 (3.90)	** < 0.0005**	31/54 (57.41)		
Days at ICU, median (IQR)	12 (4–20)	12 (5–18)	16 (2–45)	0.62	12 (4–20)		
Oxygen, N/total N (%)	168/311 (54.02)	91/157 (57.96)	77/154 (50.00)	0.17	52/54 (96.30)	116/257 (45.14)	** < 0.0005**
Days with oxygen supplement, median (IQR) N = 168	5 (2–11)	8 (3–12)	4 (2–7)	**0.0042**	10 (5–19)	4 (2–8)	** < 0.0005**
Mechanical ventilation, N/total N (%)	24/311 (7.72)	20/157 (12.74)	4/154 (2.60)	**0.001**	24/54 (44.44)		
Days with mechanical ventilation, median (IQR) N = 24	14 (7–21)	13 (7–19)	28 (7.5–46.5)	0.49	14 (7–21)		
Length of hospitalization, median (IQR)	4 (0–9)	5 (1–13)	3 (0–7)	**0.01**	18 (9–27)	2 (0–6)	** < 0.0005**
Long hospitalization, N/total N (%)	83/311 (26.69)	54/157 (34.39)	29/154 (18.83)	**0.002**	45/54 (75.93)	42/257 (16.34)	** < 0.0005**
Days from onset of symptoms to admission, median (IQR) N = 292	4 (2–8)	6 (2–9)	3 (1–6)	**0.0071**	2 (1–7)	5 (2–8)	**0.04**
Readmission, N/total N (%)	62/311 (19.94)	28/157 (17.83)	34/154 (22.08)	0.35	12/54 (22.22)	50/257 (19.46)	0.64
Symptoms N/total N (%)							
Fever	216/311 (69.45)	125/157 (79.62)	91/154 (59.09)	** < 0.0005**	45/54 (83.33)	171/257 (66.54)	**0.015**
Runny nose	17/311 (5.47)	10/157 (6.37)	7/154 (4.55)	0.62	3/54 (5.56)	14/257 (5.45)	1
Cough	175/311 (56.27)	112/157 (71.34)	63/154 (40.91)	** < 0.0005**	35/54 (64.81)	140/257 (54.47)	0.16
Sore throat	30/311 (9.65)	18/157 (11.46)	12/154 (7.79)	0.34	4/54 (7.41)	26/257 (10.12)	0.80
Shortness of breath/dyspnea	176/311 (56.59)	105/157 (66.88)	71/154 (46.10)	** < 0.0005**	47/54 (87.04)	129/257 (50.19)	** < 0.0005**
Headache	60/311 (19.29)	24/157 (15.29)	36/154 (23.38)	0.071	11/54 (20.37)	49/257 (19.07)	0.83
Muscle aches/myalgia	63/311 (20.26)	34/157 (21.66)	29/154 (18.83)	0.54	9/54 (16.67)	54/257 (21.01)	0.47
Diarrhea	58/311 (18.65)	30/157 (19.11)	28/154 (18.18)	0.89	8/54 (14.81)	50/257 (19.46)	0.56
Malaise	81/311 (26.05)	38/157 (24.20)	43/154 (27.92)	0.46	17/54 (31.48)	64/257 (24.90)	0.32
Nausea	43/311 (13.83)	14/157 (8.92)	29/154 (18.83)	**0.013**	3/54 (5.56)	40/257 (15.56)	0.053
Vomiting	32/311 (10.29)	15/157 (9.55)	17/154 (11.04)	0.71	2/54 (3.70)	30/257 (11.67)	0.088
Tiredness	66/311 (21.22)	40/157 (25.48)	26/154 (16.88)	0.064	16/54 (29.63)	50/257 (19.46)	0.096

The distributions of treatments, outcomes, and symptoms were presented for all hospitalized patients. Treatments, outcomes, and symptoms are compared between patients admitted in waves 1 and 2 in addition to patients with and without critical COVID-19 disease

Bold p-values specify significance < 0.05

*IQR* interquartile range, *N* number, *ICU* intensive care unit

The proportion of patients with critical disease during the second wave was smaller than during the first wave (5.84% vs. 28.66%). Only six patients were admitted to the ICU during the second wave compared to 25 patients during the first wave. Similarly, significantly more patients died during admission or within 30 days after their diagnosis during wave 1 than during wave 2. The median time from onset of disease symptoms to hospital admission and the median length of hospitalization were shorter during the second wave. Furthermore, the number of patients receiving mechanical ventilation and the median number of days with oxygen supplementation were significantly lower during the second wave. Significantly more patients experienced fever, dyspnea, and cough during wave 1 compared to wave 2 whereas fewer patients experienced nausea.

Patients with critical disease were admitted faster and for a longer time than patients without critical disease. For patients with critical disease, the median time from onset of symptoms to admission was two days (IQR: 1–7), and the median length of hospitalization was 18 days (IQR: 9–27). Most patients with critical disease received oxygen supplementation and 44% underwent mechanical ventilation. Dyspnea and fever were also highly prevalent among patients with critical disease and present in more than 80% of the patients.

### Sources of infection

Details on the sources of infection are presented in Table 4.

**Table 4:** Presentation of the source of COVID-19 infection

		Wave	
Source of infection N/total N (%)	All patients (N = 311)	Wave 1 (N = 157)	Wave 2 (N = 154)
Infected at home	48/311 (15.43)	15/157 (9.55)	33/154 (21.43)
Infected outside the home	38/311 (12.22)	12/157 (7.64)	26/154 (16.88)
Infected at hospital	17/311 (5.47)	15/157 (9.55)	2/154 (1.30)
Infected at an institution	18/311 (5.79)	8/157 (5.10)	10/154 (6.49)
Unknown source of infection	190/311 (61.09)	107/157 (68.15)	83/154 (53.90)

The sources of infection are presented for all patients and patients admitted in waves 1 and 2, respectively

The sources of infection were known for approximately 40% of all patients in the present study. The sources of infection were known for 46% of the patients in wave 2 and 32% of the patients in wave 1. In general, most infections were acquired at home. We observed a difference in the sources of infection between waves 1 and 2. During the first wave approximately 10% were infected at the hospital compared to 1% during the second wave. In contrast, more patients were infected at home during the second wave.

## Discussion

In the comparison of the clinical presentation of hospitalized patients admitted during the first and second waves of COVID-19 in the western part of Denmark, we found a milder course of disease during the second wave period from July to December 2020 compared to the first wave period from March to June 2020. Fewer patients experienced critical disease during wave 2. Furthermore, patients admitted during wave 2 were hospitalized and received oxygen supplementation for a shorter time than patients from wave 1. In addition, fewer patients received mechanical ventilation and experienced symptoms such as fever, cough, and dyspnea. Despite a milder course of disease, patients hospitalized in wave 2 were admitted significantly sooner after the onset of symptoms than those hospitalized in wave 1. Patients admitted during wave 2 were mostly infected with COVID-19 at home, and only a few were infected at the hospital. We found no significant differences in sociodemographics, lifestyle information, or laboratory data in the comparison of patients admitted during the first and second waves of COVID-19 in 2020.

Recent studies have compared the characteristics of hospitalized patients from different waves of COVID-19 [2, 3, 14–16, 19–22]. However, the studies reported different measurements, inclusion criteria, and aims. To our knowledge, only a few studies have performed a comprehensive comparison of the clinical characteristics with the inclusion of laboratory data [14, 15]. Likewise, comparisons of the sources of infection from different waves of COVID-19 have only been investigated to a limited degree [21].

In agreement with our study, studies from Spain, Japan, and Iran indicated a milder course of disease during the second wave period [2, 3, 20]. These studies likewise reported a lower mortality among hospitalized patients with COVID-19 in the second wave than in the first wave [2, 3, 20]. Similar to our study, Iftimie et al. reported a shorter hospitalization during the second wave, Saito *et* al. reported a shorter period from disease onset to admission during the second wave, and Jalali et al. reported a decreased percentage of individuals admitted to the ICU during the second wave [2, 3, 20]. In contrast, a Swiss study reported similar in-hospital mortality and risk of ICU admission in waves 1 and 2 [16].

Although not statistically significant, we observed a tendency toward higher CRP and creatinine levels among patients admitted in wave 1 than in wave 2. This finding is in line with higher CRP and creatinine levels among patients with a critical disease since more patients from wave 1 experienced critical disease. In comparison, Mollinedo-Gajate *et* al. also reported a higher CRP level among patients with a critical or fatal disease and a higher level of CRP during the first COVID-19 wave [15]. To our knowledge, the present study is the first to compare levels of creatinine between different waves of COVID-19.

Likely explanations for a milder course of disease during the second wave compared to the first wave of COVID-19 are numerous. First, vulnerable and elderly persons were more likely to be exposed to the virus at the beginning of the pandemic compared to wave 2. Different restrictions, prohibitions, and orders were applied in Denmark from mid-March 2020 to control the spread of the disease and to protect vulnerable and elderly persons. Thus, we assume that a larger percentage of the population was at risk of critical disease during the first wave of COVID-19. Second, the initial treatment of COVID-19 was mostly symptomatic, however, as the clinical management was refined over time, this could possibly have improved the outcome for those hospitalized later during the pandemic. Refined clinical management could likewise have resulted in a reduced length of hospitalization in wave 2 compared to wave 1. Third, potential changes in SARS-CoV-2 genomic variations from wave 1 to wave 2 could have influenced the disease severity among patients, as SARS-CoV-2 genomic variations have been associated with the mortality rate of COVID-19 [23]. Finally, the Danish testing strategy may have increased the number of patients with a milder course of disease during wave 2 in the present study. The testing strategy was adjusted several times during 2020. In the beginning of the pandemic, the total Danish testing capacity was limited but increased considerably over time. During wave 2, all hospitalized patients were tested before or during admission. In contrast to wave 2, secondary COVID-19 diagnoses were not necessarily registered during wave 1. Thus, the study population from wave 2 may contain more patients with milder courses of disease.

Interestingly, patients were admitted sooner in wave 2 than in wave 1, increasing the likelihood of timely treatment. Potentially, this could have decreased the number of individuals experiencing critical disease.

Most studies investigating risk factors for COVID-19 were conducted during the beginning of the pandemic [11, 24–26]. Thus, a secondary aim of our study was to identify risk factors for critical COVID-19 disease in a study population with the inclusion of patients from two different waves in 2020. As expected, we identified older age, male sex, smoking, comorbidities, fever and dyspnea as risk factors for a critical COVID-19 disease course. Furthermore, we observed significantly increased levels of CRP and creatinine, and lower hemoglobin levels among patients with a critical disease. Our results are thus in line with the results from a systematic review including 207 studies from the spring of 2020 [27]. Izcovich et al. identified sociodemographic characteristics (age, male sex and smoking), comorbidities, and increased levels of CRP and creatinine as prognostic factors for mortality and/or severe COVID-19 disease [27]. Another study investigating the COVID-19 mortality rate in 16 countries likewise found increased risk of COVID-19 death among elderly persons in addition to a higher risk in males than in females [28].

In the beginning of the pandemic, the transmission of severe acute respiratory syndrome coronavirus 2 (SARS-CoV-2) was high in the service area of the RHWJ compared to the rest of Denmark [6]. Data on sources of infection from this study did not provide an explanation of this matter. Unfortunately, the sources of infection were unknown for the majority of patients. Interestingly, however, we observed a change in the sources of infection comparing information from the first and second wave. Approximately 10% of the patients were infected at the hospital during wave 1. In contrast, only a few individuals were infected at the hospital in the second wave. A study of the prevalence of SARS-CoV-2 antibodies among hospital employees during wave 1 showed increased seroprevalence among employees at the RHWJ compared to other hospitals [29]. This may have contributed to the increased numbers of hospital acquired COVID-19 infections in the beginning of the pandemic. Furthermore, the testing strategy may have increased the risk of infections at the hospital during wave 1.

By inclusion of all hospitalized COVID-19 patients in a geographical area comprising 5000 km^2^ and a population of approximately 286,000 inhabitants, our study population is representative of the most severely affected COVID-19 individuals in the western part of Denmark in 2020. The Danish healthcare system is universal, publicly funded, and based on the principles of free and equal access to healthcare, including tests, for all citizens. A key strength of this study was that all patients were included consecutively and followed from admission to discharge. Furthermore, all patients were included in the study before the Danish population was offered vaccination against COVID-19. Overall, these factors help eliminate selection bias.

The present study has some limitations due to the study design and number of cases. We performed a single-center study, which affects the external validity. Furthermore, the retrospective study design affects the available data, especially the documentation of sources of infection. The present study did not include individual information on the pharmalogical treatment. In general COVID-19 patients at RHWJ were treated using thromboprophylaxis. During wave 2 the pharmacological treatment also comprised remdesivir, dexamethasone, and tocilizumab. Thus patient outcomes may have been affected by differences in the pharmacological treatment. Wave periods of the pandemic have evolved at different times on continents/countries during the pandemic [30]. In the beginning of the pandemic, the number of COVID-19 admissions at RHWJ was high [6]. However, only 12 patients from our study were hospitalized in the period from July to October. Hereafter, the number of admitted COVID-19 patients increased and remained high at the end of 2020. We chose July 1st as a pragmatic cut-off date between the first and second waves of COVID-19 and in agreement with a study by Iftimie et al. [3]. Importantly, however, the conclusions of the study remained the same if October 1, was chosen as cut-off date. The inclusion of patients in the present study ended December 31, 2020. Thus, hospitalized patients from the second half of wave 2 in 2021 were not included in this study. Potentially, the selection of the cut-off date between the wave periods and the lack of some patients from the second half of wave 2 in 2021 could have introduced minor biases in the study. However, we have no reason to assume differences in symptoms and/or severity for patients admitted during the first and second half of wave 2.

## Conclusion

Our study is a new comprehensive investigation comparing demographical, clinical, and laboratory characteristics in addition to sources of infection between two COVID-19 waves in 2020. The results bring new knowledge to the field and confirm and qualify previous findings. In conclusion, the course of disease was worse among patients hospitalized in wave 1 than in wave 2. The length of hospitalization was significantly longer in wave 1 and more patients experienced critical disease. However, we observed no significant differences in baseline or laboratory characteristics among patients admitted in wave 1 compared to wave 2. Presumably, the health conditions of patients upon admission and treatment during admission were better in wave 2 compared to wave 1. In general, a large percentage of patients were infected with COVID-19 at home, and most hospital-acquired COVID-19 infections were observed during the first wave. In agreement with other studies including patients from wave 1, we identified higher age, male sex, smoking, comorbidities, fever and dyspnea as risk factors for critical COVID-19 disease. Furthermore, we observed significantly increased levels of CRP and creatinine, and lower hemoglobin levels among patients with critical disease.

Further studies investigating the laboratory characteristics of COVID-19 patients and sources of infection are warranted. In addition, future studies comparing risk factors for critical diasease in different waves of COVID-19 could be interesting.

## Data Availability

Public access to electronic medical records is not available due to Danish legislation. Restrictions apply to the availability of these data. Thus, these data are not public available. Data are, however, available upon reasonable request and with permission from the Central Denmark Region (Forskningsprojekter@rm.dk).

## Abbreviations

BMI: Body Mass Index
COVID-19: Coronavirus disease 2019
N: Counts
CRP: C-reactive protein
ICU: Intensive care unit
IQR: Interquartile ranges
PCR: Polymerase chain reaction
RHWJ: Regional Hospital West Jutland
SARS-CoV-2: Severe acute respiratory syndrome coronavirus 2
